# Point prevalence and risk factors for pressure ulcers in hospitalized adult patients: a cross-sectional study

**DOI:** 10.31744/einstein_journal/2024AO0811

**Published:** 2024-08-28

**Authors:** Bruna Andrade de Oliveira, Flávia Cristina Zanchetta, Beatriz Barbieri, Carolina Akmiy Schiezaro Falcioni, Eliana Pereira de Araújo, Maria Helena Melo Lima

**Affiliations:** 1 Faculdade de Enfermagem Universidade Estadual de Campinas Campinas SP Brazil Faculdade de Enfermagem, Universidade Estadual de Campinas, Campinas, SP, Brazil.; 2 Hospital de Clínicas Universidade Estadual de Campinas Campinas SP Brazil Hospital de Clínicas, Universidade Estadual de Campinas, Campinas, SP, Brazil.

**Keywords:** Pressure ulcer, Prevalence, Risk factors

## Abstract

**Objective:**

To estimate the point prevalence of and risk factors associated with the development of pressure ulcers at a university hospital in Brazil.

**Methods:**

This study was conducted on 196 participants using a structured questionnaire, physical examination of the skin, and the Braden scale. The Mann-Whitney U, χ^2^, or Fisher’s exact tests were used to compare the participants and the associations of variables with pressure ulcers. A modified multivariate Poisson regression model was built considering the presence of pressure injuries and the independent variables.

**Results:**

The point prevalence of pressure ulcers was 10.71% and was significantly associated with less than 12 years of schooling (p=0.0213), use of antihypertensive drugs during hospital stay (p=0.0259), diagnosis of systemic hypertension (p=0.0035), and *diabetes mellitus*. Lower scores on the Braden scale (p=0.0001) were positively associated with the presence of pressure ulcers. Furthermore, cardiovascular disease (p=0.0267) and diaper use (p=0.0001) were associated with the presence of pressure ulcers. Moreover, they were also associated with prolonged hospital stay, advanced age, less than 12 years of schooling, use of antihypertensive drugs, hypertension, diabetes, and lower Braden scale scores.

**Conclusion:**

Health professionals should be aware of the risk factors associated with pressure ulcers, evaluate patient skin daily, and offer prevention. Our findings support the need to allocate resources for the prevention and treatment of pressure injuries.

## In Brief

Oliveira et al. showed that the occurrence of pressure ulcers was positively associated with the length of hospital stay, advanced age, incomplete high school education, use of antihypertensive drugs, diagnosis of hypertension and diabetes, and lower Braden scale scores.

## Highlights

Excess moisture can lead to maceration and contribute to skin breakdown.

Superficial pressure ulcers were the most common and can be easily prevented.

Healthcare professionals should be aware of PU risk factors, evaluate skin daily, and offer prevention.

## Graphical abstract

**Figure:**
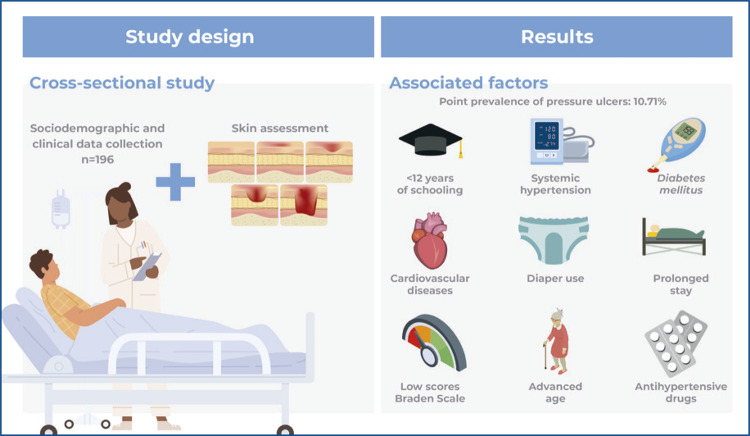

## INTRODUCTION

Pressure ulcers (PUs) are conceptualized as the breakdown of skin integrity and/or underlying tissue as a result of pressure or pressure associated with shear.^(1,2)^ Hospitalized patients are vulnerable to PUs due to mobility limitations and/or comorbidities.^(3)^

The most frequent risk factors for PUs include: advanced age; immobility; limited sensory perception/perfusion, most often due to poorly controlled diabetes;^(4,5)^ non-blanchable PUs; incontinence; malnutrition; vasopressor drugs;^(5)^ lower Braden scale scores;^(6,7)^ prolonged hospital stay;^(6-8)^ and intensive care unit (ICU) admission.^(7,9,10)^

The prevalence of PUs varies among clinical settings and countries. A systematic review and meta-analysis of 39 studies with more than 2,500,000 participants from 19 countries identified a combined prevalence of 12.8%.^(11)^ Therefore, more than one in 10 hospitalized adult patients was affected by PUs.^(11)^ In Brazil, the prevalence of PUs is estimated to be between 10.8% and 25.6%, with a higher prevalence in ICU settings and a lower prevalence in older people living in long-term care institutions.^(12-14)^ Another Brazilian study conducted at a teaching hospital with two different periods of data collection, but in the same year, identified a PU prevalence of 10.8%.^(15)^

Pressure ulcers prevention can save costs for healthcare facilities and improve the quality of nursing care. Hence, systematic risk assessments and preventive measures are effective in reducing the occurrence of hospital-acquired PUs.^(16)^ Instruments such as the Braden scale, which is accepted worldwide, have also been validated in Brazil,^(17)^ and are designed to assist in the early identification of individuals at risk of developing PUs.^(18)^

In addition, the best practices are widely disseminated through clinical guidelines.^(19)^ The international strategy for the prevention and treatment of PUs produced by the European Pressure Ulcer Advisory Panel (EPUAP) and National Pressure Injury Advisory Panel (NPIAP)^(20)^ disseminate this information worldwide.

Therefore, it is important to know the prevalence of PUs in each health unit so that care related to prevention can be better managed in relevant sectors.

## OBJECTIVE

To estimate the point prevalence and risk factors for the development of pressure ulcers in a university hospital in Brazil.

## METHODS

### Study design and setting

This cross-sectional study was conducted over a 1-week period in September 2022 in adult inpatient units and ICUs of a quaternary care university hospital in Brazil. The hospital has 18 adult inpatient units and eight ICUs. Healthcare is provided via the Brazilian Public Health System (SUS - *Sistema Único de Saúde)*.

To maximize uniformity in reporting, the team was trained in order to standardize information and was instructed on how to use the platform and enter the collected data, perform physical examination of the skin, measure and predict anthropometric data (weight and height), apply the Braden scale and, and if PU was detected, classify the injury according to the NPIAP.^(20)^ After training, the authors worked in pairs and collected data together so as to increase its reliability.

### Participants

The study population comprised 397 participants, including 343 adult inpatients and 54 ICU patients. The sampling error was estimated to be 4%, and the significance level was set at 5%. The largest sample size included 196 participants with different prevalence rates. The sample size was divided according to the number of beds available for adult patients in the ICU. The remaining units were randomly drawn at http:// www.sortear.net.

The inclusion criteria were patients aged 18 years or older who were admitted to the hospital until data collection. Bone marrow transplantation, hematology, and psychiatric wards were excluded considering their specificity and patient characteristics.

### Data collection and identification of pressure ulcers

Data were collected from September 18 to September 25, 2022, using a questionnaire that contained sociodemographic and clinical data (age, sex, length of hospital stay, comorbidities, medical devices used, and anthropometric data). Regular physical examinations of the skin were performed. The skin was palpated and inspected in the cephalocaudal direction in order to check for the presence of PUs. The content validity of the questionnaire was confirmed by a panel of experts consisting of five members from the School of Nursing at the University of Campinas. Minor changes were made based on recommendations from the experts.

The risk of developing PUs was assessed using the Braden Scale. Information about hospital admission dates and comorbidities was obtained from the patient charts. PUs were classified according to the NPIAP as stage I, II, III, IV, unstageable PU, or deep tissue PU,^(20,21)^ and all grading was performed by at least two researchers. The research team visited each patient once during the data collection period.

The Braden scale, adapted and validated for Brazil,^(17)^ was used to complement the clinical data and evaluate the risk of developing PUs. Six risk factors were considered: sensory perception, skin moisture, mobility, activity, nutrition, friction, and shearing.^(22)^ These factors were scored from 1 to 4, except for friction and shear, which were scored from 1 to 3. The scale ranged from 6 to 23 points, and the lower the value, the higher the risk for PUs. Injuries were also classified using a risk score: severe risk (≤9 points), high risk (10-12 points), moderate risk (13-14 points), low risk (15-18 points), and no risk (≥19 points).^(23)^ Anthropometric data (weight and height) were used to calculate body mass index (BMI), which was classified as: underweight (<18.5kg/m^2^), healthy weight (18.5-24.99kg/m^2^), overweight (25-29.99kg/m^2^), or obese (≥30kg/m^2^), as established by the World Health Organization.^(24)^

### Data analysis

Descriptive analyses of sociodemographic and clinical data are presented as frequencies and percentages. SAS version 9.4 (SAS Institute Inc., Cary, NC, USA) was used for all statistical analyses and the significance level was set at 5%. The Mann-Whitney U test was used to compare age and length of hospital stay between participants with and without PUs.^(25)^ Data distribution was assessed using the Shapiro-Wilk test. The χ^2^ test was utilized to evaluate the associations between the presence of PU and other qualitative variables.^(25)^ Fisher’s exact test was used for those cases in which the assumptions of the χ^2^ test were not met.^(26)^ A modified robust variance-error multivariate Poisson regression model was built,^(27)^ with PUs as the dependent variable. Independent variables included in the model were based on clinical criteria, taking into consideration previous literature and the results of the association and comparison tests, *i.e*., variables whose p-value was lower than 0.20. Moreover, age, sex, and length of hospital stay were regarded as control variables. The results show the estimates for the prevalence ratio, respective confidence intervals, and p-values.

The point prevalence was calculated as: (number of participants with a pressure ulcer/number of participants in a population at a particular point in time) × 100. Point prevalence was defined as the number of patients with PUs at a specific point in time (often on a specific day).^(11)^

### Ethical considerations

The study protocol was reviewed and approved by the Research Ethics Committee of the *Hospital de Clínicas, Universidade Estadual de Campinas* (CAAE: 36414720.8.0000.5404; # 4.384.628). Written and verbal permission was obtained from the patients and their families (in the case of patients who were unable to communicate due to sedation, intubation, or altered level of consciousness) prior to data collection in order to protect individual rights.

## RESULTS

Of the 196 participants, 26 (13.26%) were admitted to the ICU. Sociodemographic data showed that most participants were male (59.18%), Caucasian (62.76%), married (53.57%), and had less than 12 years of schooling (62.75%). The mean age of the participants was 54.75 years. Based on BMI, 13.27% of the patients were underweight, 49.49% were healthy, and 37.25% were overweight or obese.

The most prevalent comorbidities were systemic hypertension (46.15%) and cardiovascular diseases (37.50%) including acute myocardial infarction, atrial fibrillation, and heart failure. *Diabetes mellitus* (DM) accounted for 22.56% of patients, and neurological, psychiatric, pulmonary, vascular, and renal diseases accounted for less than 10%.

Forty-five patients (23.44%) self-reported being former drinkers, 53 (27.6%) were drinkers, 54 (28.13%) were former smokers, and 27 (14.06%) were smokers. Four participants did not answer questions regarding their lifestyle.

Regarding the medications used during data collection, 122 (62.24%) were antibiotics, 155 (79.08%) were anticoagulants, 182 (92.86%) were anti-inflammatory drugs, and 110 (56.12%) were antihypertensive medications.

Lower limb edema was detected in 45 (22.95%) patients, indwelling urinary catheters were present in 53 (27.04%), and diapers were worn in 78 (39.80%).

According to the Braden scale, 73 (37.24%) patients had a moderate, high, or severe risk of developing PUs.

There were 27 PUs among the 21 patients in this study, with an overall PU prevalence of 10.7%. One PU was observed in 17 (81.0%) patients, two injuries were detected in two (9.5%), and three injuries were found in two (9.5%). Regarding the classification of Pus,^(19)^ 12 (44.4%) were classified as stage I, 10 (37%) as stage II, and five (18.5%) as stage III. As for the site of the injuries, 20 (74.0%) were located in the sacral region, three (11.2%) on the calcanei, two (7.4%) on the buttocks, and two (7.4%) in the trochanteric region.

Table 1 shows the distributions of variables and their significant associations with the presence of PUs: schooling (p=0.0213), use of antihypertensive medications during hospital stay (p=0.0259), diagnosis of systemic hypertension (p=0.0035), DM (p=0.0267), cardiovascular diseases (0.0425), Braden scale score showing moderate, high, or severe risk of PUs (p=0.0001), and diaper use (p=0.0001).

**Table 1:** Associations between the variables related to the occurrence of pressure ulcers and the other qualitative variables

Variable	Pressure ulcer		p value

	No n (%)	Yes n (%)	
Schooling			
Less than 12 years	105 (85.3)	18 (14.6)	0.0213^† §^
More than 12 years	70 (95.8)	3 (4.1)	
Antihypertensive drug			
No	103 (93.6)	7 (6.3)	0.0259^† §^
Yes	72 (83.7)	14 (16.2)	
Systemic arterial hypertension			
No	100 (95.2)	5 (4.7)	0.0035^† #^
Yes	74 (82.2)	16 (17.7)	
*Diabetes mellitus*			
No	139 (92.0)	12 (7.9)	0.0267^‡ §^
Yes	35 (79.5)	9 (20.4)	
Cardiovascular disease			
No	169 (90.3)	18 (9.6)	0.0425^‡ §^
Yes	5 (62.5)	3 (37.5)	
Braden scale			
No risk/low risk	121 (98.3)	2 (1.6)	0.0001^† #^
Moderate risk/high risk	54 (73.9)	19 (26.0)	
Diaper			
No	114 (96.6)	4 (3.4)	0.0001^† #^
Yes	61 (78.2)	17 (21.8)	

^†^ χ^2^ test; ^‡^ Fisher exact test. Significance Level: ^§^ p<0,05; ^#^ p<0,0001.

Quantitative variables, such as age and length of hospital stay, associated with the presence of PUs are shown in table 2. Longer hospital stay and advanced age were positively associated with the development of PUs.

**Table 2:** Comparing participants with and without pressure ulcers according to age and hospital stay

Variable	Pressure ulcer	n	Mean (SD)	IIQ	Median (Min-Max)	p value
Hospital stay	No	175	15.7 (35.3)	12	7.0 (0.0-352.0)	0.002
	Yes	21	25.8 (23.2)	35	22.0 (1.0-96.0)	
Age (Years)	No	175	53.3 (17.3)	27	55.0 (19.0-94.0)	0.001
	Yes	21	66.2 (13.4)	18	69.0 (32.0-89.0)	

SD: standard deviation; IIQ: interquartile interval; Min-Max: Minimum-Maximum.

Sex, ethnicity, medication use, smoking, drinking, other underlying diseases, lower limb edema, BMI, and indwelling urinary catheter were assessed in search of associations with the presence of PU; however, no statistical difference was observed.

The logistic regression model demonstrated that the presence of cardiovascular disease (p=0.0448) and diaper use (p=0.0184) were associated with PU development (Table 3). The Braden scale was not included in the model because of the low frequency of PU in non-risk and low-risk patients, which would compromise the reliability of the estimates.

**Table 3:** Modified Poisson Regression for factors associated with the presence of pressure ulcers

Dependent variable	Independent variables	Prevalence ratio	95%CI		p value

			Lower limit	Upper limit	
Pressure ulcer	Antihypertensive drug (ref = No)	1.59	0.66	3.80	0.2974
	*Diabetes mellitus* (ref = No)	1.33	0.54	3.29	0.5359
	Cardiovascular disease (ref = No)	2.43	1.02	5.78	0.0448
	Vascular diseases (ref= No)	1.96	0.70	5.49	0.2014
	BMI (ref = Underweight/ Healthy)	1.08	0.46	2.51	0.8617
	Diaper (ref = No)	4.91	1.31	18.4	0.0184
	Indwelling urinary catheters (ref = No)	1.04	0.39	2.82	0.9342

Model adjusted for age, gender, and hospital stay.

95%CI: 95% confidence interval; BMI: body mass index; Ref: Reference.

## DISCUSSION

This was a cross-sectional study on the presence of PUs in patients admitted to a university hospital. This study included 196 patients, 21 of whom had at least one PU; hence, the prevalence of PUs was 10.71%. This prevalence was lower than that reported in an Irish multicenter trial, in which the prevalence of PUs was 18.5%.^(28)^ In contrast, a systematic review and meta-analysis including 2.5 million patients worldwide showed a PU prevalence of 12.8% among hospitalized adult patients.^(11)^ The difference in PU prevalence between countries may be related to regional discrepancies, specific characteristics of each unit where the patients stay, the health status of each patient, and available preventive measures.^(4,11,29,30)^

In this study, most PUs were classified as stage I or II. This indicates that PUs in advanced stages (III and IV) may be preventable if daily skin assessments are performed. Regarding the PU region, the sacral region was the most widely affected area, which is consistent with previous studies.^(11,29,31,32)^ Besides everyday skin evaluations, the Braden scale should be used for PU risk management. This scale is a global reference for identifying patients at risk of developing PU. Most studies have shown that lower Braden scale scores are significantly associated with the presence of PUs.^(4,5)^ The findings of this study concur with those reported in the literature; lower scores were associated with the development of PUs.^(11,30,32,33)^

Along with lower Braden scale scores, the use of antihypertensive medications, comorbidities such as hypertension and/or diabetes, cardiovascular diseases, length of hospital stay, older age, less than 12 years of schooling, and diaper use were associated with PUs.

With respect to comorbidities, hypertension and treatment with antihypertensive drugs in addition to *Diabetes mellitus* were positively associated with the presence of PUs. According to another study^(34)^ there is scant evidence that medication use predisposes patients to PUs. This is likely to be a surrogate indicator of an underlying disease that increases the risk of PUs. In a review article, no association was found between hypertension and PUs.^(35)^ Conversely, one study suggested that an association between PUs and cardiovascular disease occurs because of poor blood perfusion and advanced age.^(35)^ As far as DM is concerned, prolonged hyperglycemia causes microvascular complications, leading to local ischemia and delayed healing.^(29,36)^ Moreover, injuries to peripheral nerves reduce sensory perception.^(35)^

Comorbidities were associated with longer hospital stay. In this study, the length of hospital stay was associated with the development of PU, which is consistent with the findings of other studies.^(4,11,29-31,34,37)^ We found that age greater than 60 years was a risk factor for the development of PU. This could be due to poor skin status, poor nutrition, and mobility limitations, all of which predispose patients to PUs.^(34)^ A study that assessed 16 health facilities in Finland demonstrated that the prevalence of PUs increased among older patients.^(38)^

Previous studies have not found an association between the development of PUs and poor schooling among hospitalized patients, which is in agreement with our findings. According to some studies^(39,40)^ schooling may interfere with the understanding of and compliance with clinical guidelines, especially when patients are receiving home-based medical care, which differs from our study in which the patients were cared for by health professionals.

Regarding sex, studies^(34,38)^ have not provided sufficient evidence to suggest that sex is a risk factor for the development of PUs. However, a systematic review and meta-analysis showed that age and sex were predictive factors for PUs.^(11)^

In addition to sociodemographic variables, previous studies^(6,8,41)^ have positively associated individual risk factors such as being overweight, having lower limb edema, and urinary catheter use with hospital-acquired PUs. Unlike the findings reported in the literature, the present study did not find a positive association between these variables and the development of PUs; however, we found that diaper use was associated with and a risk factor for PU development. Studies have demonstrated that incontinence is a major risk factor for PUs^(34,42)^ as it exposes the skin to moisture, urine, and feces, altering the local microclimate, and thereby affecting tissue-protective factors and favoring PU development.^(35,43)^ Therefore, it may be mandatory to elaborate PU guidelines and/or protocols with specific indications for diaper use.

Finally, the point prevalence of PUs at our university hospital was consistent with previous systematic reviews.^(11)^

### LIMITATIONS

This study had a few limitations. First, the preventive measures adopted for each patient have not been described. Second, the findings provided information on a single university hospital, and the sample size, although estimated using statistical tests, was small. Therefore, these findings should be interpreted with caution when applied to global scenarios.

## CONCLUSION

In this study, the prevalence rate of pressure ulcers was lower than that reported in most studies using the same methodology. We found that superficial pressure ulcers, such as stages I and II, were the most common and can be easily prevented. Therefore, we recommend that healthcare professionals should be aware of these risk factors, evaluate patient skin daily, and offer prevention. Our findings support the need to allocate resources for the prevention and treatment of pressure ulcers.
